# Delphi survey to inform patient-reported symptom monitoring after ovarian cancer treatment

**DOI:** 10.1186/s41687-020-00237-2

**Published:** 2020-08-28

**Authors:** Leanne Shearsmith, Fiona Kennedy, Oana C. Lindner, Galina Velikova

**Affiliations:** Patient Reported Outcomes Group, Section of Patient-Centred Outcomes Research, Leeds Institute of Medical Research (LIMR) at St James’s, University of Leeds, St James’s Hospital, Level 6 Bexley Wing, Beckett Street, Leeds, LS9 7TF UK

**Keywords:** Ovarian cancer, Follow-up care, Internet, Symptoms

## Abstract

**Background:**

Increasing numbers of ovarian cancer patients are living longer and requiring regular follow-up to detect disease recurrence. New models of follow-up care are needed to meet the growing number and needs of this patient group. The potential for patient-reported outcome measures (PROMs) to capture key symptoms and online technology to facilitate long-term follow-up has been suggested.

**Objectives:**

Prior to a pilot study exploring the potential for electronic patient-reported symptom monitoring, the content of an online intervention was developed via Delphi methodology.

**Design and setting:**

A Delphi process was conducted aiming to obtain consensus amongst the clinicians and patients from 4 hospitals on the key aspects to monitor during follow-up after ovarian cancer treatment, and how to monitor them in an online intervention. A two round Delphi was conducted. Consensus was defined as at least 70% agreement.

**Results:**

Out of 43 participants, 30 (18 patients, 12 healthcare professionals) completed round 1 and 19 (11 patients, 8 healthcare professionals) completed round 2. Consensus was reached on the key symptoms to monitor, and the importance of monitoring both duration and frequency of symptoms. Opportunity for review of psychological wellbeing and holistic needs were considered important by both groups. The frequency of online questionnaire completion, timeframe for patients to reflect on (e.g. during the past X weeks), and the choice of PROMs items to monitor symptoms did not reach the consensus threshold.

**Conclusion:**

It is crucial that any intervention and the selection of PROMs is fully described to ensure transparency about the development and decisions taken. In this work, a set of key symptoms and areas to monitor were agreed, which has informed the design of an online intervention and a subsequent pilot study is now underway. The proposed model of remote follow-up using electronic PROMs could be adapted and explored in other cancer sites.

## Plain English summary

**What is the key problem/issue/question this manuscript addresses?**

With growing numbers of patients living longer following cancer treatment, new models of follow-up care are needed. Using technology, symptoms can be monitored remotely as part of long term follow-up, but this requires careful planning to ensure it meets patient needs.

**Why is this study needed?**

It is important to explore the views of patients and healthcare professionals so they can contribute to the design of any new follow-up process.

**What is the main point of your study?**

Before piloting a web-based system, we conducted a survey amongst patients and healthcare professionals to gather their views about the symptom-related questions we should ask, and how often patients should be answering these in order to manage symptoms/side effects and detect recurrence.

**Provide a brief overview of your results and what they mean.**

Patients and healthcare professionals agreed on the key symptoms to monitor, and the importance of having an opportunity to report and request support for any holistic needs following treatment. Differences of opinion were highlighted in how often symptoms should be reported. This work has informed a web-based symptom reporting system which is being piloted within a community follow-up pathway.

## Introduction

Ovarian cancer survival is improving, leading to an increase in patients on routine follow-up and growing pressure on clinics as incidence rates are expected to rise further [1]. Ovarian cancer follow-up is not protocol-driven and evidence-based best practice is yet to be established [2]. Current practice involves monitoring symptoms and serum biomarkers (CA125) through routine appointments to detect recurrence, which is likely to occur within 5 years [3]. Evidence is scarce on the value of routine follow-up for survival or quality of life [4]. Nama et al. [5] suggested that follow-up could utilise patient-reported outcome measures (PROMs) as symptoms are often present at the point of relapse. Research has also indicated that patients delay help-seeking for symptoms until their next appointment [6], and some gynaecological patients have high anxiety before/during appointments [7]. More sustainable models of follow-up and long-term symptom tracking are clearly needed to ensure care provision is commensurate with patient need, rather than a one-size-fits-all service which may detract resources from those who need them most [8].

In recent years pilot studies have explored nurse-led telephone methods [9–12]. Most have been small qualitative studies, but they illustrate positive experiences amongst early endometrial or stage I-IV ovarian cancer patients. Cox [10] notes the convenience of telephone follow-up in facilitating psychosocial support and the reassurance of continued blood tests.

Recent proliferation of modern technology means the internet offers another follow-up method [13]. Some patients prefer face-to-face appointments, but a recent UK-based randomised controlled trial illustrates the feasibility and patient value of online symptom self-reporting when clinicians are engaged [14]. Furthermore, web-mediated follow-up (supplementary, weekly) vs. routine follow-up has demonstrated increased survival/earlier relapse detection [15].

To explore the feasibility of an electronic PROMs (ePROMs) follow-up pathway, the ‘electronic Patient self-Reported outcomes to Improve cancer Management and patient Experiences’ (ePRIME) study proposed an online intervention for ovarian patients following treatment. The intervention included an ePROMs symptom questionnaire and a blood test to monitor CA125 (performed at GP, local hospital or cancer centre). The clinical team interpret these results, instead of the patient attending hospital-based appointments. Prior to piloting this pathway, decisions about the intervention content and logistics required consultation, which is an important step of any intervention development [16]. This paper describes the process undertaken to reach agreement from healthcare professionals (HCPs) and patients on the core symptoms for monitoring relapse and life after treatment, the most appropriate symptom measure/items, and the frequency of completion to inform the ePROM intervention.

## Methods

A Delphi consultation methodology was chosen as it offers an iterative consensus-based approach to reach agreement [17]; group responses are fed back to participants allowing them to re-assess their views within multiple rounds. A ‘modified’ Delphi survey [18] was undertaken which allows the initial round to be informed from group discussions/interviews or a literature review, and is less prescriptive on the number of rounds required. The survey was conducted in two iterative rounds over a 10 month period (Round 1 August 2016–February 2017, Round 2 February–April 2017, but one patient completed in June 2017).

### Participants

All doctors and clinical nurse specialists (CNS) working in the gynae medical oncology teams at the four participating hospitals were invited to complete the online survey (https://www.onlinesurveys.ac.uk/) via an email, including a unique username/password for access. Paper copies were provided on request.

Patients who were between 6 and 36 months post-treatment and attending routine ovarian cancer follow-up were consecutively invited as they could offer opinions on the proposed online intervention having experienced the follow-up process. Purposive sampling aimed to get a mixture of patient ages and time/experience on follow-up. Patients were approached by their clinical team, and if willing were provided with a study information sheet. Following verbal agreement patients were given a paper survey and a username/password if they preferred to complete the online version.

All participants were reminded that the survey was anonymous, and the survey return was taken as formal consent. One email/telephone reminder was undertaken.

The demographic details collected included patient age, time post-treatment, and whether this was their first diagnosis. HCPs age, gender, role, and years worked in speciality were collected.

### Procedure and Delphi survey content

Ethical approval was obtained from NHS Leeds West Research Ethics Committee (ref: 16/YH/0239).

Firstly, the key symptoms to monitor during ovarian cancer follow-up were explored through informal discussions with HCPs at each hospital. A literature review was conducted to identify and select six potential PROMs for this clinical group (see Additional file 1: Appendix 1 for details of the review and the measures).

**Fig. 1:**
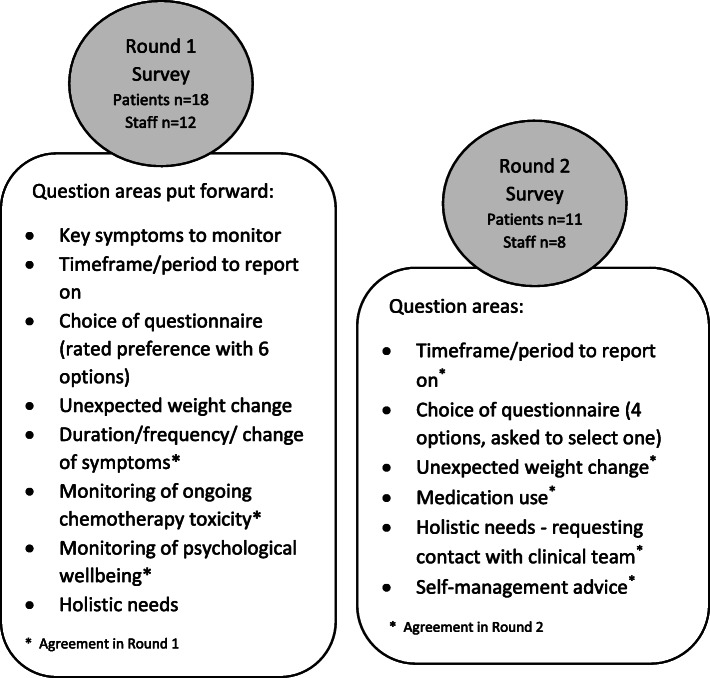
Schematic showing questions areas on the Delphi panel in each round

Figure 1 outlines the survey topics and an abridged version of each round is presented in Additional file 1: Appendix 2.

#### Round 1

Relevant symptom items from each of the six PROMs (Additional file 1: Appendix 1, Table A1) were selected and grouped, rather than presenting the full measure (see Additional file 1: Appendix 2 for an example). Then participants rated how well the overall set of items captured the key symptoms to monitor on a 7-point Likert scale (1 = not at all, 7 = very much). The survey also explored the frequency of completion in a follow-up setting, the timeframe for patients to reflect upon (e.g. during the past X weeks), whether symptom duration/frequency/change and holistic needs should be captured.

#### Round 2

The results from round 1 (aggregated percentages from HCPs, patients, overall) were presented, and further questions asked where opinion was diverse or new aspects had emerged since round 1.

### Statistical analysis

There is no universal agreement in Delphi methodology on the percentage that constitutes ‘consensus’, with recommendations ranging from 51% to 80%, but Humphrey-Murto et al. (2017) suggested 70% is a typical cut-off [19] and therefore this was defined a priori as the proposed target level. A lack of consensus was defined as percentages below 70% in round 2.

The proportion of each response was also explored across the participant groups (patients, HCPs, overall). For items on a Likert scale (e.g. round 1-PROMs rating 1–7), mean scores were calculated for each measure for each group/overall, and the highest scoring PROMs were presented in round 2.

Descriptive analysis was performed using Microsoft Excel 2013.

## Results

### Round 1

Forty-three participants (28 patients/15 HCPs) were approached, and 30 responded (69.8% response rate), including 18/28 patients (64.3%) and 12/15 HCPs (80%). Table 1 illustrates the demographic characteristics in both rounds. Half the patients (*n* = 9) were 6–12 months post-treatment, whilst others were 1–3 years post-treatment, and for most patients it was their first diagnosis (*n* = 14). The HCPs included at least one consultant and CNS from each hospital, but more from the largest hospital (site 1).

**Table 1:** Demographic details of the Delphi participants in each round

**HCPs**		**Round 1** **(*n*** **= 12)**	**Round 2** **(*n*** **= 8)**
*Age*	26–45	5	4
	46–55	7	4
*Gender*	Male	3	2
	Female	9	6
*Professional role*	Consultant	7	5
	CNS	5	3
*Time working in ovarian cancer*	1–5 years	2	2
	6–10 years	6	4
	10+ years	4	2
*Hospital site*	Site 1	5	5
	Site 2	2	1
	Site 3	2	1
	Site 4	3	1
**Patients**		**Round 1** **(*n*** **= 18)**	**Round 2** **(*n*** **= 11)**
*Age*	18–45	3	2
	46–55	2	1
	56–65	4	4
	66–75	8	4
	76+	1	0
*How long since last treatment?*	6–12 months	9	7
	1–3 years	9	4
*First diagnosis or relapse?*	First	14	7
	Relapse	4	4
*Hospital site*	Site 1	7	4
	Site 2	4	3
	Site 3	2	0
	Site 4	5	4

The key symptoms agreed were: abdominal pain/discomfort, abdominal swelling/bloating, nausea, vomiting, appetite loss, change in bowel habit, urinary symptoms, shortness of breath, fatigue, swollen legs and unexpected weight change. Table 2 summarises the percentage agreement for the questions in each round.

**Table 2:** Results from each round, percentage of agreement for each area and the consequent decisions made

**Round 1** ***N*** **= 30 (12 HCPs, 18 patients)**			
**Areas where consensus reached:**	**%**	**Areas where consensus was not reached:**	**Round 1 findings (overall %, unless otherwise stated) and** ***any decisions made in italics***
*Ask how long a symptom has been present*	Yes – HCPs 100% (12/12) Patients 88.9% (16/18) Overall **93.3%** (28/30)	*Choice of PROMs:* • EORTC-OV28 • FACT-O • MDASI-OC • MOST • PRAE • PRO-CTCAE (1 = not at all, 7 = very much)	EORTC – HCPs mean 5.75; patient mean 6.12 FACT-O – HCPs mean 5.25; patient mean 5.82 MDASI-OC – HCPs mean 4.58; patient mean 5.59 MOST – HCPs mean 6.00; patient mean 6.24 PRAE – HCPs mean 6.08; patient mean 6.18 PRO-CTCAE – HCPs mean 5.08; patient mean 6.00 *MDASI-OC and FACT-O not rated highly so discarded in Round 1; PRO-CTCAE kept due to patient score.*
*Ask how frequent the symptom has been experienced*	Yes – HCPs 83.3% (10/12) Patients 88.9% (16/18) Overall **86.7%** (26/30)	*Frequency of completion* (Options: 4 monthly, 3 monthly, 2 monthly, monthly, 6 weekly, other)	4 monthly - 0 3 monthly – 36.7% (11/30) 2 monthly – 26.7% (8/30) Monthly – 20% (6/30) 6 weekly – 13.3% (4/30) Other – 3.3% 1/30 *4 monthly, 6 weekly, ‘other’ discarded in Round 1.*
*Ask if symptom has got better / worse / not changed recently*	Yes – HCPs 100% (12/12) Patients 88.9% (16/18) Overall **93.3%** 28/30)	*Timeframe/period of reporting* (Options: Last day, last week, last 2 weeks, last month, since last reported)	Last week – 23.3% (7/30) Last 2 weeks – 23.3% (7/30) Last month – 23.3% (7/30) Since last reported – 23.3% (7/30) Last day – 6.7% (2/30) *Last day option discarded in Round 1.*
*Ask about ongoing toxicity to chemotherapy*	Yes – HCPs 75% (9/12) Patients 83.3% (15/18) Overall **80%** (24/30)		
*Ask questions to screen for psychological wellbeing*	Yes – HCPs 91.6% (11/12) Patients 72.2% (13/18) Overall **80%** (24/30)	*List of non-physical holistic needs* – select which items should be presented to patients to indicate if they are concerned (e.g. travel insurance, sexual, emotional)	50% (15/30) felt should include all areas Highest individual items were: Emotional 40% (12/30), Psychological 33.3% (10/30) 2 patients said ‘none of areas’
		*Weight change question*	Highest scoring option “Have you been concerned about changes in your weight?” HCPs 75% (9/12) Patients 61.1% (11/18) Overall 66.7% (20/30) *Other options discarded in Round 1.*
**Round 2** ***N*** **= 19 (8 HCPs, 11 patients)**			
**Areas consulted on:**	**Round 2 findings (overall %, unless otherwise stated)**	**Final decisions made:**	
*Frequency of completion* • 3 monthly • 2 monthly • Monthly	• 3 monthly **68.4%** (13/19; 6/11 patients, 7/8 HCPs) • 2 monthly 15.8% (3/19; 2/11 patients, 1 HCPs) • monthly 15.8% (3/19) – all patients	Did not quite meet 70% consensus overall, although 87.5% HCPs agreed. Final decision to complete **3 monthly** - with anytime access for patients allowing them to report sooner/more frequently if needed	
*Choice of PROMs (tick ONE option)* • EORTC-OV28 • MOST • PRAE • PRO-CTCAE	• 10.5% (2/19; 1 patient, 1 HCPs) • 26.3% (5/19; 1 patient, 4 HCPs) • **42.1%** (8/19; 5 patient, 3 HCPs) • 21.1% (4/19; all patients)	Did not reach 70% consensus. Patients chose PRAE most often 5/11 (45.5%) followed by PRO-CTCAE (4/11), HCPs chose MOST 4/8 (50%), followed by PRAE. **PRAE items chosen as preferred by most overall.**	
*Timeframe/period to reflect on when answering symptom questions:*	• Last week 15.8% (3/19; all patients) • 2 weeks **47.4%** (9/19; 5 patient, 4 HCPs) • Last month 21.1% (4/19; 1 patient, 3 HCPs) • Since last reported 15.8% (3/19; 2 patient, 1 HCPs)	Did not reach 70% consensus. Both patients and HCPs chose ‘**last 2 weeks**’ most often.	
*List of non-physical holistic needs* Part 1: “Would a page like this be acceptable to you?” and Part 2: should patients be able to tick which issues they’d like specific advice/contact with their clinical/CNS about?	Yes to part 1 – Overall 89.5% (17/19) HCPs 100% (8/8) Patients 81.8% (9/11) Yes to part 2 – HCPs 100% (8/8) Patients 90.9% (10/11) Overall 94.7% (18/19)	**Include holistic needs and ask patients if they need advice/ contact with their clinical/CNS team in relation to these**	
**Further new areas consulted on:**			**Final decisions made:**
*Provision of self-management advice* (Options: yes, unsure, no, other)	*Yes –* Overall 26.3% (5/19) HCPs 62.5% (5/8) Patients 0	*Other –* 2 patients chose ‘other’ indicating they’d want option to speak to nurse over the phone.	Split between ‘yes’ amongst HCPs, and no/unsure amongst patients. Free text comments suggested that at least contact details should be provided. **Final decision to include self-management advice for mild symptoms, in particular telephone contact details for CNS team.**
	*No –* Overall 31.6% (6/19) HCPs 25% (2/8) Patients 36.4% (4/11)	*Unsure –* Overall 31.6% (6/19) HCPs 12.5% (1/8) Patients 45.5% (5/11)	
*Ask about medication use in relation to symptoms experienced*	*Yes –* HCPs 75% (6/8) Patients 90.9% (10/11) **Overall 84.2%** (16/19)		**Yes include**
*Would this question ‘Have you been concerned about changes in your weight?’ be acceptable?*	*Yes –* HCPs 100% (8/8) Patients 81.8% (9/11) **Overall 89.5%** (17/19)		“Unexpected” weight changes were always the focus of this item, and emphasised again in Round 2 free-text comments - **item changed to “Have you been concerned about unexpected changes in your weight?”**

Overall 93.3% felt it was important to monitor the duration and change in symptoms, and 86.7% agreed that symptom frequency should be explored. Eighty percent felt ongoing chemotherapy toxicity and psychological wellbeing were important.

Consensus was not achieved for how frequently patients should complete the online questionnaire, the timeframe/period that patients reflected upon (… past X weeks), and the specific holistic needs question (e.g. travel insurance, emotional). Similarly, the choice of which PROMs to use did not reach consensus, but two options (MDASI-OC; FACT-O) were removed as these were endorsed the least. HCPs also scored the PRO-CTCAE low but it was retained into round 2 due to patient endorsement.

### Round 2

15/18 patients wished to be contacted for round 2, and 11/15 completed (73.3%). All HCPs were re-contacted regardless if they had completed round 1. One nurse was no longer working in the speciality, but 8/14 HCPs completed (57.1%), including 5 consultants and 3 CNS (5/8 were from site 1, with one representative from the other 3 sites, see Table 1). Overall 19/29 responded (65.5%).

Three new areas were consulted (Table 2), namely: the provision of self-management advice (62.5% HCPs yes, but 45.5% patients unsure), asking about medication use (84.2% overall), and weight changes (89.5% overall).

The frequency of completion fell short of reaching agreement with 68.4% endorsing 3-monthly reports. However, the acceptability of including a checklist of holistic needs (e.g. emotional, financial, sexual, work/employment, family issues) was agreed by 100% HCPs and 81.8% patients.

Sufficient consensus was not reached on the timeframe (… past X weeks) or the preferred PROMs to use. Given the time constraints a third round could not be undertaken. Discussions were held within the project steering committee, and as around half (47.4% overall) chose ‘ … past 2 weeks’ this was selected. PROMs opinions varied widely for three measures across the participant groups (PRAE 42.1% - 5 patients/3 HCPs; MOST 26.3% - 1 patient/4 HCPs; PRO-CTCAE 21.1% - 4 patients) (Table 2). Further consultation selected the PRAE as this measure was endorsed most overall, particularly by patients who were prioritised as the intended online system users.

**Fig. 2:**
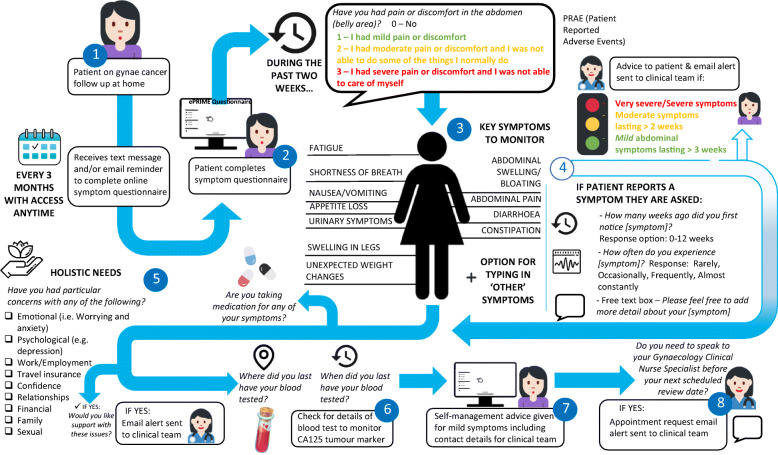
Overview of the final symptom monitoring intervention

Figure 2 visually presents the symptom monitoring intervention.

## Discussion

This work achieved its aim of exploring the key symptoms to monitor during ovarian cancer follow-up, and gathering HCP/patient views of the frequency/proposed content of an online follow-up intervention. The value of remote online methods to facilitate follow-up has become increasingly relevant during/following the COVID-19 pandemic where the reduction of face-to-face hospital-based appointments was essential. This has led to calls for rapid introduction of electronic PROMs in cancer clinical practice [20] and the continued importance of providing support for cancer survivors during/following the pandemic [21].

For most areas, 70% consensus was achieved within two rounds, but some major differences were observed within and between groups. We hope this report provides transparency of the process [16] to enable others to learn from our experiences.

Most HCPs felt 3-monthly was an appropriate frequency (in line with clinical practice), but patients were divided between monthly and 3-monthly reports. For the ePRIME pilot 3-monthly was chosen but the system is open-access should patients wish to complete earlier. This work highlights the importance and value of consulting both patient and HCPs equally when developing interventions [22, 23].

Furthermore, rather than a purely remote system whereby patients report online without clinical contact, free-text comments and discussions with both groups led to the decision that a telephone-based appointment would allow a CNS to formally review online completions and blood results. Therefore, this format is being evaluated in the ongoing pilot.

Patients and HCPs placed great value on reporting/reviewing holistic needs, adding to the evidence that holistic needs assessment is integral to delivering person-centred care [24]. Within the ePRIME system we specifically ask about non-medical holistic needs, and we would urge others to continue prioritising this important area in research and clinical practice, especially if follow-up care is being delivered remotely [23].

The overall survey response rates in both rounds were ~ 65%, and non-response/attrition reasons were not collected. Selection bias is possible as non-responders may have contrasting views of the proposed intervention. Furthermore, those who dropped out may be different, for example less older patients (66+ years) completed round 2. However individuals were consecutively approached, there was a variety of demographics and hospital sites represented across both rounds.

Consensus was not reached on the choice of which PROMs to use, with views divided across patients and HCPs. However, as discussed the level of percentage agreement is disputed in the literature, and the stability of responses through rounds may be more valuable [17]. To our knowledge there is no official guidance for how to proceed in such circumstances. In hindsight a third round or a formal consensus meeting inviting all participants may have been beneficial [22], but with limited time/resources this was resolved through discussion within the project steering committee [25]. It may be that Delphi methodology is not the most appropriate method of choosing between similar validated measures.

## Conclusions

It is imperative that intervention development and the selection of PROMs for trials/research is explicitly described to ensure transparency about the decision-making and confirm the appropriateness and content validity of the intervention [16]. The views of patients and HCPs have informed the development of the ePRIME follow-up intervention which is being piloted in two hospitals in the North of England. If successful, the model of remote follow-up pathway and the ePROM system could be adapted to other cancer sites.

## Supplementary information

**Additional file 1: Appendix 1.** Literature review & identification of selected PROMs measures. **Appendix 2.** Example of questions included in each round.

## Data Availability

The anonymised datasets collected during the current study are stored at the Patient Centred Outcomes Research group. These datasets are not publicly available, but the corresponding author may consider reasonable requests.

## Abbreviations

CNS: Clinical nurse specialist
ePRIME: Electronic Patient self-Reported outcomes to Improve cancer Management and patient Experiences
ePROMs: Electronic patient-reported outcome measures
GP: General practitioner
HCPs: Healthcare professionals
PRAE: Patient Reported Adverse Event
PROMs: Patient-reported outcome measures
PRO-CTCAE: National Cancer Institute Patient-Reported Outcomes Version of the Common Terminology Criteria for Adverse Events
