# Supplementary material for: Delphi survey to inform patient-reported symptom monitoring after ovarian cancer treatment
Source: J Patient Rep Outcomes. 2020 Aug 28;4:71. doi: 10.1186/s41687-020-00237-2 (PMC7453693; doi:10.1186/s41687-020-00237-2)
Supplement: Supplementary file 1 — Additional file 1: Appendix 1. Literature review & identification of selected PROMs measures. Appendix 2. Example of questions included in each round. [file 41687_2020_237_MOESM1_ESM.docx]

**Appendix 1 – Literature review & identification of selected PROMs measures**

A rapid scoping review was conducted aiming to identify potentially suitable ovarian cancer PROMs that could be used to monitor patients’ symptoms after treatment. These measures were presented to experts in the Delphi consultation process.

The review extended the literature review by Nama et al. [1] who explored the use of PROMs in follow-up care and the systematic review by Efficace et al [2] who explored randomised control trials that included a PRO measure. Both of these reviews were undertaken in gynaecological cancer specifically and searched up to 2012, therefore our search strategy focused on 2012-2016.

We searched MEDLINE, Web of Knowledge, and the Cochrane reviews database, drawing on the search strategies outlined by Nama et al. [1], and combining MESH terms “Ovarian Neoplasm” AND “Questionnaire”, “Quality of Life”, “Outcome Assessment (Health care)”.

In particular the review was focused on ovarian-gynaecological cancer specific instruments, but two generic item banks (PRO-CTCAE, PRAE) were included. This work identified 10 potential PROMs measures, which were reviewed and the individual items compared with the key symptoms that had been identified during initial discussions with the clinical teams. Some measures were discounted due to not covering all the key symptoms (e.g. City of Hope QOL-Ovarian questionnaire does not cover bloating, shortness of breath, swollen legs) and others had no published record of validation (e.g. Public Health England NHS Living with and beyond ovarian cancer questionnaire, which also drew on other validated measures).

To make the Delphi questionnaire survey process less burdensome for all, we selected 6 of the validated measures/ item banks (Table A1 shows the selected measures and some examples of the items) and presented the items that were specific to monitoring ovarian cancer patients within the Delphi survey.

Table A1 – Ovarian PROMs measures presented in the Delphi survey

| **Measure** | **Example ‘bloating’ questions/responses:** |
| --- | --- |
| European Organisation for Research and Treatment of Cancer: Quality of Life Questionnaire ovarian specific module (EORTC-QLQ-OV28) [3] [Greimel et al. 2003] | *Did you have a bloated feeling in your abdomen / stomach?* Responses: 1 -Not at all, 2 - A little, 3 - Quite a bit, 4 - Very Much |
| Functional Assessment in Cancer Therapy Scale-Ovarian (FACT-O) [4] [Basen-Engquist et al., 2001] | *I feel bloated.* Reponses: 0 -Not at all, 1 - A little bit, 2 - Somewhat, 3 - Quite a Bit, 4 - Very Much |
| M.D. Anderson Symptom Inventory-Ovarian Cancer (MDASI-OC) [5] [Sailors et al., 2013] | *Your feeling bloated at its WORST?* Responses: 0 - Not present to 10 - As bad as you can imagine |
| Measure of Ovarian Symptoms and Treatment (MOST) [6] [King et al., 2014] | *Abdominal swelling, bloating and/or fullness.* Responses: 0 - No trouble at all, 2 - Mild, 5 - Moderate, 8 - Severe, 10 - Worst I can imagine |
| National Cancer Institute Patient-Reported Outcomes Version of the Common Terminology Criteria for Adverse Events (PRO-CTCAE) [7] [Basch et al. 2014] | *In the last 7 days, how OFTEN did you have bloating of the abdomen (belly)* Responses: Never / Rarely / Occasionally / Frequently / Almost constantly  *In the last 7 days, what was the SEVERITY of your bloating of the abdomen (belly)* *at its WORST*  Responses: None / Mild / Moderate / Severe / Very severe |
| Patient Reported Adverse Event (PRAE) items [8] [Holch et al. 2016] | *Did you feel bloating (uncomfortable fullness) of the tummy (abdomen)?*  Responses:  0 - No  1 - I had mild bloating of the tummy but I was able to eat and drink the same amount and type of food as usual  2 - I had moderate bloating of the tummy and I ate or drank less than usual  3 - I had severe bloating of the tummy and I was not able to eat or drink |

1. Nama, V., A. Nordin, and A. Bryant, *Patient-reported outcome measures for follow-up after gynaecological cancer treatment.* Cochrane Database Syst Rev, 2013(11): p. CD010299.

2. Efficace, F., et al., *Patient-reported outcomes in randomised controlled trials of gynaecological cancers: investigating methodological quality and impact on clinical decision-making.* Eur J Cancer, 2014. **50**(11): p. 1925-41.

3. Greimel, E., et al., *An international field study of the reliability and validity of a disease-specific questionnaire module (the QLQ-OV28) in assessing the quality of life of patients with ovarian cancer.* Eur J Cancer, 2003. **39**(10): p. 1402-8.

4. Basen-Engquist, K., et al., *Reliability and validity of the functional assessment of cancer therapy-ovarian.* J Clin Oncol, 2001. **19**(6): p. 1809-17.

5. Sailors, M.H., et al., *Validating the M. D. Anderson Symptom Inventory (MDASI) for use in patients with ovarian cancer.* Gynecologic oncology, 2013. **130**(2): p. 323-328.

6. King, M.T., et al., *Development of the Measure of Ovarian Symptoms and Treatment Concerns: Aiming for Optimal Measurement of Patient-Reported Symptom Benefit With Chemotherapy for Symptomatic Ovarian Cancer.* International Journal of Gynecological Cancer, 2014. **24**(5): p. 865-873.

7. Basch, E., et al., *Development of the National Cancer Institute’s Patient-Reported Outcomes Version of the Common Terminology Criteria for Adverse Events (PRO-CTCAE).* JNCI Journal of the National Cancer Institute, 2014. **106**(9): p. dju244.

8. Holch, P., et al., *Asking the right questions to get the right answers: using cognitive interviews to review the acceptability, comprehension and clinical meaningfulness of patient self-report adverse event items in oncology patients.* Acta Oncologica, 2016. **55**(9-10): p. 1220-1226.

**Appendix 2 – Example of questions included in each round**

***Round 1 (abridged from patient version)***

1. Are there any other symptoms you think are important to monitor in the follow-up of ovarian cancer? (Open-ended response)

1. How often do you think patients should be asked to think about and report their symptoms on the online monitoring system within the first year following active treatment?

*Responses: monthly, 6-weekly, 2 monthly, 3 monthly, 4 monthly, other (please specify)*

1. Over what time period do you think patients should be asked to think about and report having experienced any symptoms?

*Responses: in the last day, in the last week, in the last 2 weeks, in the last month, since last reported, other (please specify)*

1. EORTC-OV28 example (note this question is repeated for each of the 6 PROMs measure options):

The questions in the box below have been drawn from the EORTC questionnaires (generic and ovarian-specific). Please look at the questions in the box and then answer the specific questions that appear outside of the box underneath.


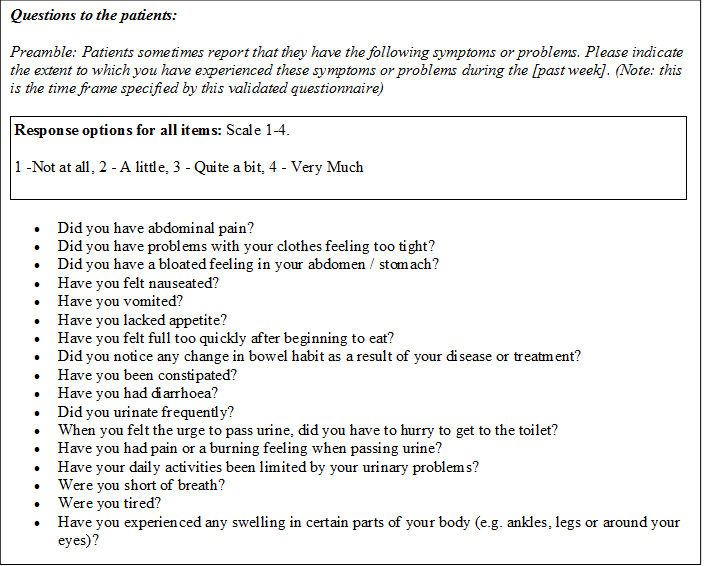


On the scale below, **please** **rate how well this questionnaire captures the symptoms which need to be monitored in ovarian cancer**, where 1=Not at all and 7=Very much

*Responses: 1, 2, 3, 4, 5, 6, 7*

Please use the space provided below to write any additional comments about this set of questions (Open-ended response)

1. Which of the following issues do you think we should ask relating to **unexpected weight changes during the last review period?** *(Select as many options as you consider important to ask)*

*Responses: Have you been concerned about changes in your weight, Has weight loss been a problem for you, Has weight gain been a problem for you, None of the above.*

If you have any comments relating to your choice above please specify here (Open-ended response)

1. Do you think it's important to ask patients how long a symptom has been present?

*Response: Yes, No, Don’t know*

If yes, please could you suggest how we might ask this question? (Open-ended response)

1. Do you think patients should be asked how frequent the symptom has been experienced?

*Responses: Yes, No, Don’t know*

If yes, please could you suggest how we might ask this question? (Open-ended response)

1. Do you think patients should be asked whether the symptom has got better/worse/not changed recently?

*Responses: Yes, No, Don’t know*

If yes, please could you suggest how we might ask this question? (Open-ended response)

1. Do you think this online monitoring system should routinely ask patients about ongoing symptoms that are related to their chemotherapy/treatment (e.g. numbness/tingling in feet or hands, hair loss, leg cramps)?

*Responses: Yes, No, Don’t know*

Please provide any comments to explain your response (Open-ended response)

1. Do you think this online monitoring system should routinely ask questions about emotional distress / psychological wellbeing (e.g. anxiety / depression)?

*Responses: Yes, No, Don’t know*

Please provide any comments to explain your response (Open-ended response)

1. We understand that the current pathway offers/aims to allow the opportunity for the patient to raise/discuss other, **non-physical holistic needs**. Which of the following aspects, if any, do you think should be presented to the patients to allow them to indicate if they are concerned about any of these issues (*Select as many options as you consider important to ask*)

*Response checkboxes: Emotional (e.g. worrying and anxiety), Confidence, Work/Employment, Financial, Travel Insurance, Family, Psychological (e.g. depression), Relationships, Sexual, All of the above, Other (please specify), None of the above*

Please take this opportunity to write any additional comments you may have (Open-ended response)

***Additional questions asked in Round 2 (abridged from patient version)***

*Note, some of the questions above were resurveyed in round 2 but where possible presenting only the most popular rated response option choices from round 1.*

*Below are the additional questions asked specifically in Round 2.*

1. Choice of PROMs measure: Through Round 1 we have been able to discard two questionnaires (FACT-O; MDASI-OC) which were not rated highly by either the clinicians or patients. This leaves us with the **4 questionnaire options now presented below**. Below we also present the percentage (%) overall (clinicians and patients) and separately for patients in terms of the Round 1 ratings of each questionnaire, and provide some examples of the comments received from clinicians and patients. **We are now asking you to select which ONE questionnaire from the 4 options above you prefer for patients to report their symptoms whilst in follow-up in the forthcoming ePRIME project. After looking at the Round 1 findings/comments above, please select your preferred questionnaire from the list below:**

*Response Options: Option 1: EORTC-OV28, Option* 2: MOST, *Option 3: PRAE, Option 4: PRO-CTCAE*

Please write below WHY you have chosen the option you have selected above (Open-ended response)

1. Weight question: Would using this question (‘Have you been concerned about changes in your weight?’) be acceptable to you?

*Responses: Yes, No, Unsure*

1. Medication use question: Do you agree that a question about medication use related to symptoms experienced should be asked?

*Responses: Yes, No, Unsure*

1. Holistic needs question: At this stage we are considering the presentation on the system of a list of issues (such as those specified above), and allowing patients to tick the one’s they are concerned about and/or also indicate which they **would like advice on** may be a way for those wanting support/advice to communicate with their clinical team/nurse specialists, who could then get in touch. For those with no issues or not wanting support they would not have to select any. Would a page on the online system like this (described above) be acceptable to you?

*Responses: Yes, No, Unsure*

1. Do you think patients should be able to tick **BOTH** which ones they have concerns about **AND** separately tick those they’d like specific advice/contact with their clinical/clinical nurse specialist about?

*Responses: Yes, No, Unsure, Other (please explain)*

1. Self-management advice question: We would now like you to indicate below whether you think automated self-management advice should be included on the ePRIME system.

*Responses: I think specific self-management advice should be provided on the system, I do not think specific self-management advice should be provided on the system, I am unsure, Other (please specify)*
